# Environmental Impact Assessment in the Former Mining Area of Regoufe (Arouca, Portugal): Contributions to Future Remediation Measures

**DOI:** 10.3390/ijerph18031180

**Published:** 2021-01-28

**Authors:** Nuno Durães, Luís Portela, Sara Sousa, Carla Patinha, Eduardo Ferreira da Silva

**Affiliations:** GeoBioTec—Department of Geosciences, University of Aveiro, Campus de Santiago, 3810-193 Aveiro, Portugal; luispedroportela@ua.pt (L.P.); sarasousaani@gmail.com (S.S.); cpatinha@ua.pt (C.P.); eafsilva@ua.pt (E.F.d.S.)

**Keywords:** contamination, arsenic, cadmium, mine, soils, surface waters, PTEs, sequential selective chemical extraction, modified degree of contamination

## Abstract

The W-Sn Regoufe mine, closed since the 1970s, was once intensively exploited for tungsten concentrates. Throughout its activity, considerable amounts of arsenopyrite-rich mine wastes were produced and, to this day, are still exposed to weathering conditions. Thus, this work aims at assessing soil contamination, using a combination of chemical, physicochemical and mineralogical analyses and sequential selective chemical extraction of the main potentially toxic elements (PTEs) in topsoils. Results show that Regoufe soils are enriched in most of the PTEs associated with the ore assemblage, but As and Cd contents far outstrip both international and national guidelines. The estimated contamination factor reveals that 67% of soil samples are classified as highly to ultra-highly contaminated. Similar distribution patterns, with the main focus around the unsealed mine adits, are observed when spatially projecting the modified degree of contamination (*m*C*_d_*) and arsenic contents. Fe-oxyhydroxides and organic matter demonstrate to have a preponderant role in the retention of Cd and As. In fact, despite the high PTE contents in soils, local surface waters are characterised by low metal(loid) contents and nearly neutral pH, with PTE concentrations below national thresholds for irrigation waters.

## 1. Introduction

Metal(loid)s are the most toxic inorganic pollutants, the reason why they are often referred to as potentially toxic elements (PTEs). Although the sources of these elements can be either natural or anthropogenic [1,2,3], the contributions to the environment from these last ones far exceed those by natural routes [1]. A typical case is mining activity, where high amounts of metal(loid)-hosting minerals are exposed to the oxidising and weathering conditions of the surface, causing the dispersion of metal(loid)s that can reach several environmental compartments and cause their contamination [4]. These processes are particularly more relevant in abandoned mines, where the lack of environmental mining regulations and the uncontrolled disposal of mine tailings with low cohesion facilitate the dispersion and mobility of contaminants to the surrounding areas [5,6,7,8]. Since these contamination effects may persist for long decades, or even centuries [9,10], the study of these processes at such areas continues to be of high relevance, not only to help solve several current problems, but also to prevent new cases of environmental contamination caused by mines. This aspect is particularly important since, currently, there is an increase in the demand for new mineral deposits or the re-exploitation of the existing ones, including those of low-grade ore. This scenario may be accompanied by an increase in severe contamination cases, as the economically viable exploitation of low-grade ore deposits requires greater refinement than the ore-rich ones.

In Portugal, intense mining activity was developed all over the country between the second half of the 19th and 20th centuries. The majority of these mines closed either by depletion of mineral reserves or by economic unfeasibility of the exploitation, which resulted in cases of widespread mine abandonment [11]. Unfortunately, proper remediation measures were often not emplaced, and to this day, the negative effects of ore extraction and beneficiation still persist in multiple abandoned mining sites. Taking that into consideration, in the last decades, research teams have made significant efforts to characterise and assess the extension of the lingering contamination, ultimately aiming at helping decision makers [12,13,14]. Indeed, some attempts to remediate this legacy have been made; however, the remediation actions were not always best suited or yielded insufficient results [15,16]. Thus, it is important to conduct thorough investigations and obtain quality data using various approaches.

One of the cases of abandonment is the underground W-Sn mine of Regoufe, intensely exploited during World War II for tungsten concentrates and closed since the 1970s. This mine is located in a rural area of Arouca County and may pose an environmental risk due to the unconfined tailings that remain there, but is also a risk to the local populations’ health, since agriculture is one of the subsistence activities of its inhabitants and there is no public water supply.

Therefore, after almost 50 years of the abandonment of mining activities, it is important to assess the potential contamination processes that persist in the area. To achieve this main goal, the following tasks were performed: (a) the determination of the physicochemical parameters and chemical contents of PTEs in soils and surface waters from the surrounding area of the Regoufe mine, (b) the spatial distribution mapping of the main PTEs and (c) the determination of PTEs availability in soils in order to estimate the potential risks for local populations.

## 2. Geological Setting

**Figure 1:**
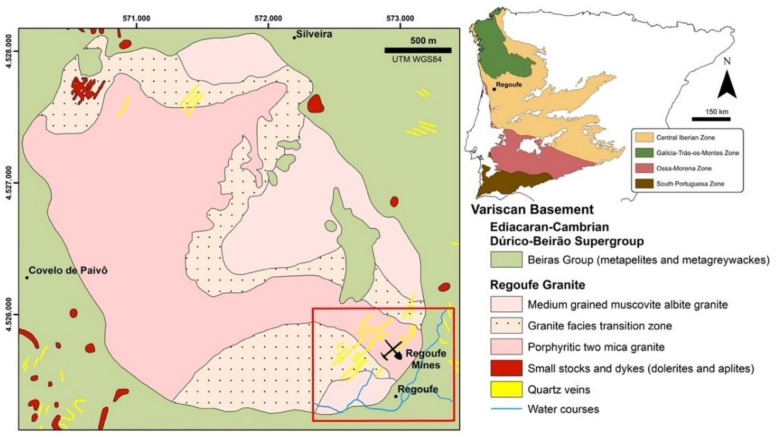
Location and geological sketch map of the Regoufe massif (modified from Vriend et al. [23]). Inset on the right was modified from Jacques et al. [27].

The Regoufe mining area (Figure 1) is located in the centre of Portugal, at the parish of Covelo de Paivô (Arouca County). This area is within the Central Iberian Zone (CIZ), one of the geotectonic domains of the Hesperian Massif [17]. The terrains of the CIZ are dominated by pre-Ordovician metasedimentary units consisting mainly of thick alternations of shales and metagreywackes, the Dúrico–Beirão Supergroup [18], which were intruded by granitic rocks during the final stages of the Hercynian orogeny [19].

The W-Sn ore deposit of Regoufe is installed in the southeast (SE) rim of a subcircular granite massif with the same name, which occupies an area of about 6 km^2^ (Figure 1). Generically, the Regoufe granite is described as a muscovite-albite porphyritic granite [20,21], with a whole-rock Rb-Sr age of 280 ± 8 Ma [22]. Although muscovite is a major constituent of the granite, it changes the composition from a medium-grained muscovite-albite granite (in the northern and eastern parts of the batholith) to a tourmaline-bearing porphyritic two-mica granite (particularly in the western sector) through a non-uniform mineralogical transition zone [23]. This granitic intrusion induced a medium- to low-grade contact metamorphism aureole in the country metasedimentary rocks of the Dúrico–Beirão Supergroup (mainly composed by phyllites and metagreywackes), which is materialised by the occurrence of biotite and phyllitic material in the low-grade regional metamorphic rocks [21]. The final stages of the granitic intrusion were characterised by hydrothermal processes that led to the precipitation of W-Sn ores in the quartz veins [21,24] and also caused hydrothermal alteration in both granite [21,24] and metasedimentary rocks [25].

The mineralisation of Regoufe consists of wolframite (the most abundant ore), cassiterite and sulphides, namely arsenopyrite, pyrite and sphalerite. Quartz, muscovite, and in minor amounts beryl and apatite, are the main gangue constituents [20,23,26].

## 3. Materials and Methods

### 3.1. Sampling

**Figure 2:**
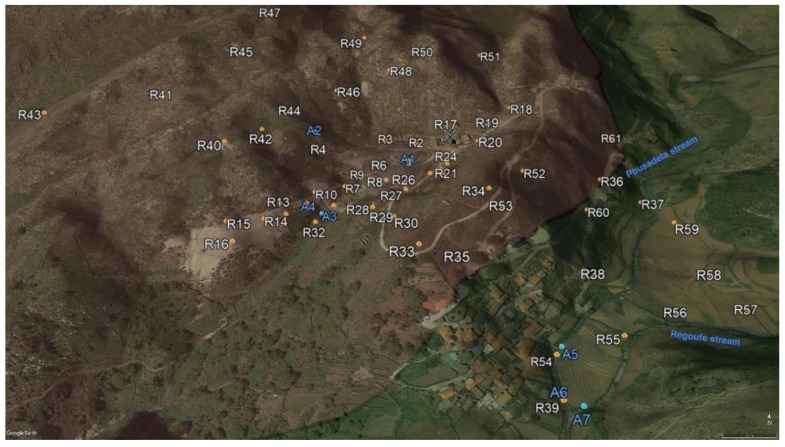
Location of the soil (white) and water (blue) sampling points. The black line marks the limit between the Regoufe granitic massif to the west (see-through pink colour) and the metasedimentary rocks to the east (see-through green colour).

A total of 61 topsoil (R1–R61) and 10 surface water (A1–A10) samples were collected from Regoufe mine precincts and surroundings (Figure 2). The soils comprised samples developed on both granite (51 samples) and metasedimentary (10 samples) bedrocks. The sampling procedure entailed the collection of ca. 2 kg of superficial debris-free soil samples (~10–20 cm depth), which were stored in individual polyethylene bags. The surface water samples were collected from the two main streams of the area (Regoufe and Pousadela streams) and from mine drainages during two sampling campaigns (wet/winter and dry/summer seasons). In the summer campaign (dry season), only three sites were sampled; thus, surface water samples A8, A9 and A10 were collected at the same locations as A1, A6 and A7, respectively. The site selection was governed by the representativeness and typology of those sampled in the first (wet season) campaign, as well as water availability. The water samples were stored in acid-washed 1 L Teflon bottles and kept refrigerated in a cooler box with ice during the transport to the laboratory.

### 3.2. Sample Processing

Water samples were filtered using 0.45-μm-pore-size nitrocellulose filters under vacuum conditions, through a Millipore^®^ sterifil aseptic system. Two subsamples (50 mL each) were reserved in free-metal falcons. One of those subsamples was destined for cation analysis and was acidified with 0.5 mL of ultra-pure HNO_3_, while the other, non-acidified, was reserved for the determination of anions. The samples were stored in a freezer until the respective analyses were carried out.

The soil samples were dried in a thermostatically controlled oven at 40 °C until no mass changes occurred. Subsequently, the samples were disaggregated, sieved through a 2 mm stainless-steel mesh and quartered to obtain two representative subsamples. One of those subsamples was again sieved using a 63 μm stainless-steel mesh, from which an aliquot was separated. The remaining <2 mm fraction underwent quartering operations in order to be split into three representative subsamples. One of those aliquots and the <63 μm fraction were pulverised in a mechanical agate mill until extremely fine and homogeneous powders were achieved for chemical and mineralogical analysis, respectively. The other <2 mm portions were reserved for physicochemical measurements and sequential selective chemical extraction (SSCE). The amounts of sample required for each analytical procedure were obtained by successive quartering of the previously separated subsamples.

### 3.3. Analysis

#### 3.3.1. Determination of Soil Mineralogy

The mineralogical content of 21 representative samples (<63 μm fraction) was determined by X-ray diffraction (XRD) at the Department of Geosciences of University of Aveiro (DGeo-UA) using a Phillips X’Pert Pro X-ray diffractometer (Almelo, The Netherlands) operating at a current intensity of 20 mA and a voltage of 40 kV, equipped with a nickel filter and a CuKα anode (λ = 1.5405 Å). The diffractograms were obtained over the 2*θ*° range from 4° to 70°, through a counting step of 0.02° per second.

#### 3.3.2. Physicochemical Parameters of Soils and Waters

The physicochemical parameters (pH, electrical conductivity (EC) and temperature) of waters were recorded at each sampling site. Temperature and pH measurements employed a pH meter (HI 8014, HANNA Instruments), previously calibrated with two standard solutions (pH 4.01 and 7.01 at 25 °C), while the EC was determined using a calibrated (1413 μS cm^−1^ at 25 °C) Cole–Parmer conductivity meter (1481-50 model).

The pH and EC of soil samples were measured in 1_W_:5_V_ suspensions of 5 g of soil in 25 mL of distilled water (pH_w_). Additionally, the soil pH was also determined in a 1_W_:5_V_ suspension of 5 g of soil in 25 mL of CaCl_2_ (0.01 M) solution (pH_CaCl2_). The measurements were done after 5 min stirring followed by a 24 h resting period [28]. These measurements were performed by using the previously described pH and conductivity meters and according to the same calibration steps.

The cation-exchange capacity (CEC) was determined according to the International Organization for Standardization - ISO 13536 [29]. In general, this method consists of soil sample (5 g) saturation with buffered (HCl 2 mol L^−1^) barium chloride (BaCl_2_ 2 mol L^−1^) solution by shaking. After this procedure, the supernatant is separated by centrifugation; the soil sample is then washed with distilled water and again centrifuged to separate the soil from the supernatant. Next, 30 mL of magnesium sulphate solution (MgSO_4_ 0.02 mol L^−1^) is added to the soil cake and then shaken, which causes the precipitation of barium (as insoluble barium sulphate) present in the solution and it is adsorbed on soil particles, while the exchangeable sites of soil particles are occupied by Mg. The mixture is then centrifuged, and the CEC is determined based on the excess of Mg in the supernatant solution. The quality of the obtained results was assessed through the analysis of 3 blanks and duplicates of 10% of the sample set. The relative standard deviation (RSD) varied between 1% and 5%.

Soil organic matter (SOM) contents were determined by loss-on-ignition at 430 °C during 16 h, after previous drying of soil samples at 105 °C for 24 h, and according to the method described by Schumacher [30]. Aside from the whole sample set, SOM contents were also determined on 10% duplicate samples, yielding acceptable RSD values ranging from 0.5 to 12.7%.

All the physicochemical parameters were determined in the DGeo-UA laboratories.

#### 3.3.3. Chemical Analysis of Soils and Waters

Chemical analyses of waters were performed at DGeo-UA. The anions (Cl^−^, SO_4_^2−^ and NO_3_^−^) were determined by ion chromatography using a Dionex 2000i chromatograph, while the concentration of HCO_3_^−^, also taken as the alkalinity value, was ascertain by volumetric titration (50 mL of sample) using H_2_SO_4_ (0.16 N). Major and trace cation concentrations were obtained by inductively coupled plasma–mass spectrometry (ICP-MS) on an Agilent Technologies 7700Series mass spectrometer (Tokyo, Japan). Random duplicates were analysed to attest for data quality. Uncertainties of these analysis were <6% for trace elements and between 2% and 7% for the major elements.

Multi-element chemical analyses of soil samples were carried out at ACME Analytical Laboratories (Vancouver, BC, Canada). A sample weight of 0.25 g was heated to fuming in a tri-acid mixture (HF–HClO_4_–HNO_3_) and taken to dryness. Subsequently, the residue was dissolved in HCl. Resulting solutions were then analysed by ICP-MS. The data quality was assessed using analytical results of certified reference materials (STD OREAS45E and STD OREAS25A-4A), blanks and random duplicate samples. The results were within the 95% confidence limits of the recommended values given for the certified materials. Depending on concentration levels of the analysed elements, the RSD values of duplicates were less than 10%, with the exception of Ca and W, showing an RSD of 16% and 32%, respectively.

#### 3.3.4. Sequential Selective Chemical Extraction (SSCE)

Ten representative samples (R1, R9, R19, R24, R32, R41, R50, R54, R57and R61), either collected in the granite and metasedimentary basements or under greater or less influence of tailings, were chosen for the SSCE methodology in order to ascertain the main support phases of the most concerning metal(loid)s in the study area. The SSCE procedure followed the main steps proposed by Cardoso Fonseca and Martin [31] and Cardoso Fonseca et al. [32], with the incorporation of a previous additional step to the original sequence. Thus, the process involved 7 sequential steps of increasing chemical strength for dissolution of the metal(loid) bond to (F1) exchangeable ions (bioavailable fraction), (F2) acid-soluble forms, (F3) Mn-oxides, (F4) amorphous Fe-oxyhydroxides, (F5) organic matter and sulphides (partially), (F6) crystalline Fe-oxides and (F7) resistant minerals (such as silicates and some oxides and sulphides). The reagents used in each step were (F1) distilled water, (F2) ammonium acetate (1 M NH_4_OAc; pH 4.5), (F3) hydroxylamine hydrochloride (0.1 M NH_2_OH HCL; pH 2), (F4) Tamm solution (0.175 M (NH_4_)_2_ C_2_O_4_−0.1 M H_2_C_2_O_4_; pH 3.3) under darkness conditions, (F5) hydrogen peroxide (H_2_O_2_ 35%), (F6) Tamm solution (0.175 M (NH_4_)_2_ C_2_O_4_−0.1 M H_2_C_2_O_4_; pH 3.3) under UV radiation and (F7) aqua regia (HCl−HNO_3_).

The concentrations of the metal(loid)s dissolved in each extraction solution were determined by ICP-MS (Agilent Technologies 7700Series; Tokyo, Japan), and the recoveries (i.e., the ratio between the sum of the concentrations obtained at different steps of the extraction and the amounts obtained by tri-acid digestion) were within 80–120%. SSCE as well as the chemical analysis of the extraction solutions were carried out at DGeo-UA.

### 3.4. Data Processing and Statistical Approaches

Principle component analysis (PCA) was used to facilitate dimensional reduction, examination and pattern identification in bulk elemental data in topsoils from the Regoufe mining area. In this work, PCA incorporated 20 variables: Al, Ca, K, Mg, Na, P, Ti, Fe, Mn, W, Sn, As, Be, Bi, Cd, Cu, Pb, Sb, U and Zn. For the principal components (PCs) to be retained after PCA, two criteria were defined: (a) the retained PCs account for at least 70% of the total cumulative variance, and (b) eigenvalues were >1.

Geological and geochemical maps were elaborated using ArcGIS software, version 10.7.1. Regarding the latter, spatial distribution maps for the most concerning PTEs were obtained by inverse distance weighted (IDW) interpolation. This method was also applied to illustrate the spatial pattern of the modified degree of contamination.

## 4. Results and Discussion

### 4.1. Soils

The mineralogical analysis allowed the identification of quartz, plagioclase, muscovite and minor amounts of K-feldspar as the main minerals present in soil samples collected under either granitic or metasedimentary bedrock. Kaolinite, gibbsite, smectite and scorodite were identified as secondary phases. This mineralogical assemblage points to the host rocks and the gangue minerals of the mineralised veins as the main sources of the inorganic fraction of these soil samples. Overall, the analysed samples can be divided into four main groups: (a) those consisting of quartz and minor amounts of muscovite and plagioclase, collected over metasedimentary bedrock; (b) quartz- and plagioclase-rich, sampled around the main deposits in small tailings on the slope flanks; (c) main tailing samples, with quartz and feldspar alteration phases; and (d) samples collected in granitic steeply sloped sites, abundant in primary minerals and secondary (supergenic) mineralogical phases (kaolinite, gibbsite and scorodite). The abundance of alteration phases in the last two groups is likely associated with the higher specific surface area of the primary minerals of the main tailings, a consequence of ore beneficiation, and with the relief-induced constant exposure of the granite substrate and continuous removal of deposited materials.

The ore minerals were not detected in XRD patterns likely due to (a) the high intensity of the maximum peaks of the most abundant minerals; (b) the fact that some of those ore minerals, such as the sulphides, are easily oxidised and give rise to secondary minerals (e.g., scorodite); and/or (c) the migration of metal(loid)s in solution to other sites.

The determined soil pH_CaCl2_ values (pH 3.40−6.00; Table 1) showed that samples are within the range of ultra-acid to moderately acid [33], with 50% of samples showing extremely acid pH values (pH median 4.30; Table 1). The pH_w_ (data not presented) can exceed the pH_CaCl2_ values by more than one unit. Although a ΔpH > 0 indicates that the cation-exchange capacity is higher than the anion-exchange capacity [34], the pH measurements performed in calcium chloride solution are preferable as it is less affected by the soil electrolyte solution concentration, particularly for soils with low EC values, as is the case. In fact, the EC values of soil samples are very low (< 158 μS cm^−1^; Table 1), typical of non-saline soils [33], and contrary to what would be expected, since low pH values generally promote the dissolution of mineral phases and the increase of ions in solution. This can be, in part, explained by the high amounts of quartz in the soil samples collected near the tailings, as such mineral is characterised by a low adsorption capacity.

On the other hand, despite SOM being highly variable (0.14–49%; Table 1), corresponding to soils with low to high contents of organic matter [35], more than 50% of the sample set revealed low contents of SOM (median 7.6%; Table 1). Organic matter has several key functions in soil, affecting both chemical and physical properties, but of greater relevance is its capacity to bind both nutrients [36] and trace elements [37]. Considering that the CEC (values varying from 0.16 to 48 cmol^+^ kg^−1^) shows a good correlation with SOM (*r* = 0.92; *p* < 0.05), this indicates that soil organic matter is mainly responsible for the sequestering of exchangeable cations on the studied soils, while secondary minerals, such as clay minerals and oxyhydroxides, seem to have a residual participation in this process.

**Figure 3:**
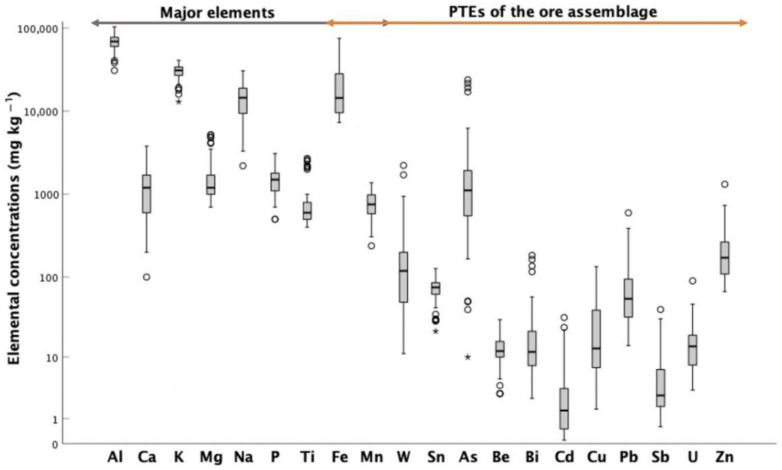
Box plots for major elements and potential toxic elements (PTEs) of the ore assemblage, displaying elemental concentration ranges. Circles and asterisks represent outlier and extreme values, respectively.

Descriptive statistics of the compositional spectrums of ore assemblage PTEs found in soil samples are presented in Table 1. In addition, for better visual inspection, the concentration ranges of both the PTEs and the major elements are illustrated in Figure 3 (box plots). As expected, in general, the concentrations of major elements were greater than PTEs and had a smaller range of variation. Calcium, Na and Fe were those with a greater concentration range, probably because of multi-sources and/or due to the greater susceptibility to weathering of their hosting minerals. Oppositely, with the exception of Sn, the PTEs showed large content intervals and a greater number of maximum anomalous values, indicating uneven and punctual dispersion. Among these elements, arsenic stands out for its remarkably high amounts and high toxicity [41].

To understand how anomalous the contents of the selected PTEs in the soil surrounding the Regoufe mine are, their concentrations were compared with the local background values (Table 1). Considering that the sampling grid of this work covered soil samples developed under granites and metasedimentary rocks, the local geochemical backgrounds (GB_local_) were assumed to be represented by the average between the geochemical backgrounds of soils from granitic (GB_gr_) and metasedimentary (GB_m_) areas.

**Figure 4:**
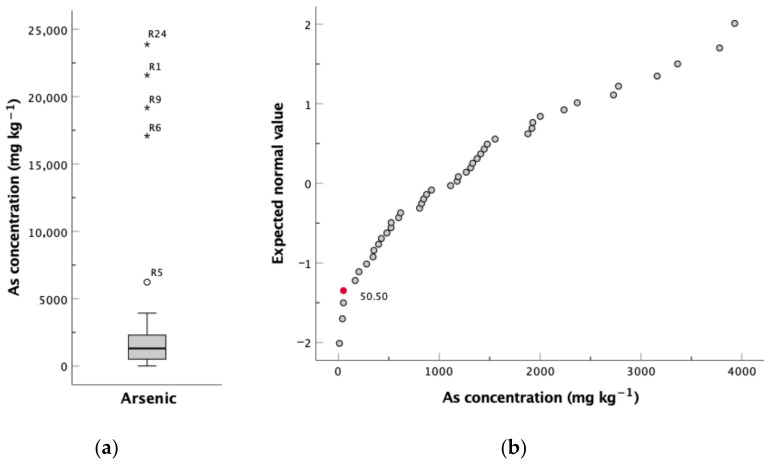
(**a**) Box plot for the concentration of arsenic in the soils developed over granite bedrock and (**b**) normal quantile–quantile (Q-Q) plot of the eliminated outliers for arsenic (red circle represents the first inflection point).

The GB_m_ values were obtained from Fonseca [38], concerning the study of another W-Sn mine (Rio de Frades mine) in the vicinity, where the mineralised veins are installed in the same metasedimentary units that crop out in the Regoufe area. The GB_gr_ values were determined according to Zhou and Xia [42], exclusively taking into account samples collected over granitic bedrock. Essentially, the method consisted of the graphical inspection of the experimental data, with the goal of distinguishing between geogenic concentration values and anthropogenically influenced samples. Accordingly, box plots were elaborated for selected elements to detect anomalous values, represented outside of the whiskers, which were removed from the dataset before the forthcoming step (Figure 4a). Outlier-free normal quantile–quantile plots (Q-Q plots) were inspected to identify breaks and inflection points, which imply the presence of different processes [42]. As illustrated in Figure 4b, the arsenic Q-Q plot undergoes inflection at 50.50 mg kg^−1^, which was assumed to represent the natural soils’ geochemical background value. In the same reasoning, geochemical background levels were determined for Bi, Cd, Cu, Fe, Mn, Pb, Sb, U, W and Zn (Table 1).

As can be seen from the data presented in Table 1, the GB_local_ values of the PTEs associated with ore paragenesis show an enrichment in relation to the geochemical background concentrations of soils from continental Portugal [40], as well as from uncontaminated world soils [39], showing that soils from this region are naturally enriched in some of the selected PTEs. This is particularly evident for As, Bi and W, which are 3, 10 and 14 higher than the geochemical background of Portuguese soils and 7, 10 and 8 times higher than the average reference world soil concentrations, respectively. Exceptions are found for Cd, Cu, Mn and Sb, for which concentrations are lower in the GB_local_ regarding at least one of the above-mentioned reference values.

Regarding the studied soil, apart from Cu and Fe (values are below the GB_local_), all the considered elements showed average amounts exceeding 2 to 55 times their respective local geochemical background (GB_local_). Among these, special attention should be given to As (55 times higher) and Cd (12 times higher) due to their role as environmental contaminants and also for their inherent toxicity [43]. The mean tungsten concentrations are also exceedingly high, surpassing by 15 times the GB_local_. However, the toxicity and deleterious health problems caused by W are still not very well elucidated [44,45]. Since tungsten mobility tends to be higher in alkaline conditions [44,46], a scenario quite different from the acid pH values determined in the local soils, its mobilisation in the solubilised phase is likely minor (Table 1).

Considering that the wide range of concentrations for several of the analysed PTEs is indicative of dispersion and/or uneven immobilisation processes, PCA was designed to understand the associations of elements in the supergenic environment.

As can be confirmed in Table 2, the first three principal components account for 75% of the geochemical variation in the soils of the Regoufe mining area. The first principal component (PC1), which explains 38.50% of the total data variance, reveals a tightly clustered association of As, Bi, Cd, Pb, Sb, W and Zn, as well as Fe and Cu. Thus, PC1 might be interpreted as a contamination trend, as the forenamed elements are associated with the geochemical signature of the Regoufe mine paragenesis. The second component (PC2), explaining 25.50% of the total variance, is defined by Ti-Mg versus a broad trend marked by Al, K, Na, Sn, Be and Mn. Such opposing associations likely reflect the contrasting mineralogy of the biotite-rich metasediments and the feldspar-rich and tourmaline-bearing granites. Finally, the third component (PC3) explains 10.61% of the total data variance and revealed a gentle U-P association, which can be associated with the presence of secondary autunite in the mineralisation [20,26]. Notwithstanding, P is the best-explained element in this PC, pointing to additional P sources as phosphate inputs through agricultural practices or even just by the natural enrichment in organic matter of some soil samples. Although Ca is not explained by any of the three PCs, it is in PC3 where this element presents a higher value, which may indicate a slight contribution of apatite, an accessory mineral of gangue veins [20,23,26].

**Figure 5:**
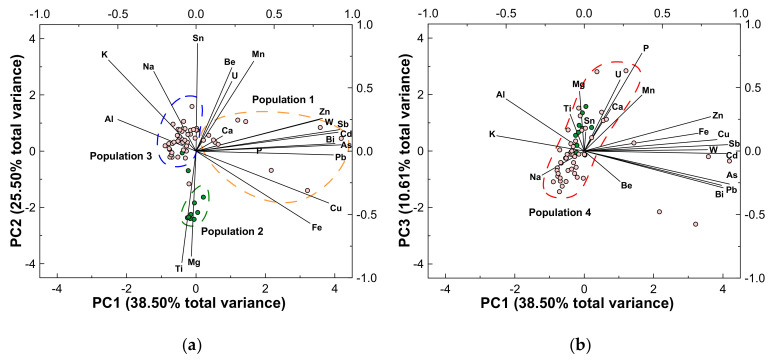
(**a**) Biplot of principal component 1 (PC1) versus principal component 2 (PC2) and (**b**) bivariate diagram with principal component 3 (PC3) as a function of PC1. Scores for samples are plotted against the bottom and left axes, while the eigenvectors for variables are plotted on the top and right axes. Pink and green circles correspond to samples collected on granitic and metasedimentary bedrock, respectively.

When plotted in the first factorial plan (Figure 5a), three populations are easily discerned: (a) Population 1 is represented by the most enriched samples of the dataset, which were collected in the proximities of the abandoned mine adits; (b) Population 2 corresponds to samples collected on the biotite-rich metasedimentary bedrock; and (c) Population 3 refers to soils sampled on the tailings and granitic basement.

When projected in the second factorial plan (Figure 5b), there is a notorious approximation of Population 2 and Population 3 of the first factorial plan, resulting in a positively sloped Population 4 that is more or less parallel to the U and P trend.

Taking into account the PTEs showing average values higher than the GB_local_ and the fact that the vast majority of them are correlated in the topsoil, the contamination factor (CF) and modified degree of contamination (*m*C*_d_*) were estimated using As, Bi, Cd, Mn, Pb, Sb, U, W and Zn, according to the methods proposed by Hakanson [47] and Abrahim and Parker [48].

The CF was calculated by dividing the mean concentration (C*_i_*) of each PTE in the soil by its corresponding background value (C*_b_*):

(1) $$\mathrm{CF}=\;\frac{C_{i}}{C_{b}}$$

Based on CF, four contamination categories can be recognised: (1) CF < 1 (low level), (2) 1 ≤ CF < 3 (moderate level), (3) 3 ≤ CF < 6 (high level) and (4) 6 ≥ CF (very high level).

The average CF values obtained for the selected PTEs showed that soils are considered moderately contaminated with Mn, Pb and Zn; highly contaminated with Bi, Sb and U; and very highly contaminated with As, Cd and W.

On the other hand, the *m*C*_d_* allows for the assessment of the overall PTEs’ soil contamination. It involved the sum of the previously computed contamination factors for each pollutant and subsequent division by the number of considered elements:

(2) $$mC_{d}=\;\frac{\left(\sum_{1\;=\;1}^{j=n}\mathrm{CF}\right)}{n}$$

where *n* is the number of analysed elements and *j* is the *j*th element. Seven *m*C*_d_* degrees can be defined and interpreted as follows: (1) *m*C*_d_* < 1.5 (none to very low), (2) 1.5 ≤ *m*C*_d_* < 2 (low), (3) 2 ≤ *m*C*_d_* < 4 (moderate), (4) 4 ≤ *m*C*_d_* < 8 (high), (5) 8 ≤ *m*C*_d_* < 16 (very high), (6) 16 ≤ *m*C*_d_* < 32 (extremely high) and (7) 32 ≥ *m*C*_d_* (ultra-high).

The *m*C*_d_* values determined for Regoufe topsoils are presented in Table 3. According to the results, about 67% of samples are classified as having a high to ultra-high degree of contamination, while only 6.5% of samples showed low to very low levels of contamination.

**Figure 6:**
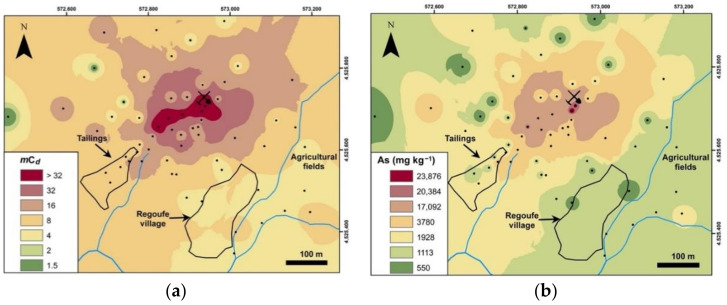
Spatial distribution maps of (**a**) the modified degree of contamination (*m*C*_d_*) and (**b**) the arsenic concentrations in topsoils of the studied area.

Given the substantial percentage of samples with high levels of contamination, it is important to visualise how this contamination is spatially distributed. In Figure 6a, the projection of *m*C*_d_* data obtained for Regoufe topsoils is presented. In this map, it is possible to observe that the main focus of contamination is located within a subcircular area surrounding the mine adits, extended either upstream or downstream, along a northwest–southeast (NW–SE) direction. Interestingly, the area where the major tailing deposit is located does not show the highest contamination levels. This might be explained by the high amounts of quartz from gangue veins, which cause a dilution effect and probably promote the migration of PTEs to deeper layers, as this process is facilitated by the low adsorption ability of quartz. In addition, the agricultural fields, which are to a certain extent shielded from the influence of the mine adits and facilities by the site’s geomorphological constraints, exhibit moderate and, punctually, high levels of contamination. The high arsenic concentrations found in the agricultural fields may be attributed to (a) inheritances from the past, when the mine was in operation and dispersion was promoted by the grinding, ore transport, etc.; (b) flooding events that enhanced transport and deposition of As-rich particulate and/or solubilised phases from other abandoned mines located upstream of this area; and/or (c) a consequence of the Regoufe natural bedrock enrichment, as verified by the higher geochemical background value of arsenic regarding the Portuguese soils’ background values. This aspect should be taken into special consideration because, as can be observed in Figure 6a,b, arsenic is mainly responsible for the *m*C*_d_* distribution pattern. As has been widely documented, arsenic can enter the food chain, representing a risk factor for human health [49,50].

**Figure 7:**
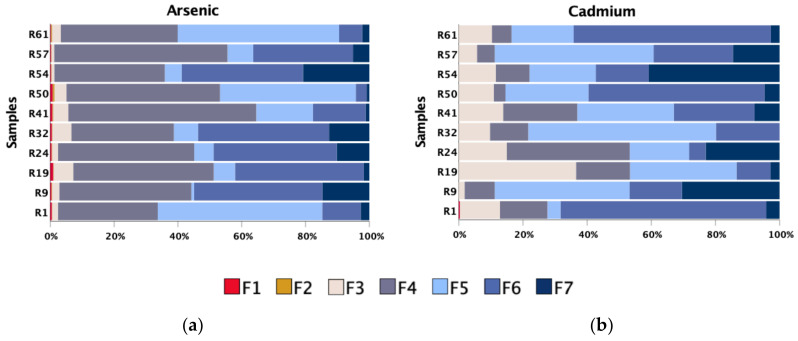
Extraction percentage of (**a**) As and (**b**) Cd obtained by sequential selective chemical extraction (SSCE) in selected soil samples: (F1) exchangeable ions (bioavailable fraction), (F2) acid-soluble forms, (F3) Mn-oxides, (F4) amorphous Fe-oxyhydroxides, (F5) organic matter and sulphides (partially), (F6) crystalline Fe-oxides and (F7) resistant minerals (such as silicates and some oxides and sulphides).

An important step in the assessment of an impacted area is to understand not only the amount and distribution of contaminants but also their main support phases and, particularly, their availability. To acquire this information, the SSCE procedure was applied to several samples. The percentage distribution, according to each inorganic/organic associated phase, for two of the most concerning PTEs (As and Cd) in the area are shown in Figure 7. As can be observed, arsenic is mainly associated with amorphous and crystalline Fe-oxyhydroxides (F4 and F6), while in some samples, particularly in those where crystalline Fe-oxyhydroxides have less expression, the association to organic matter or even to sulphides (F5) has also an important representation. The most important support phases of cadmium are crystalline Fe-oxyhydroxides (F6) and organic matter and sulphides (F5). Both elements indicate that oxidation of primary ore sulphides play a significant role and their sequestration in soil compartment is mainly ensured by secondary supergenic alteration phases and by organic matter. The percentage of As and Cd associated with the most available phases (F1 and F2) is low. However, it is important to emphasise that due to high contents of arsenic in soil samples, the amount of As extracted in the specimens selected for SSCE can reach 119 mg kg^−1^. Such concentrations are about 2.5 times higher than those of GB_local_, and almost all the analysed samples surpass the value for uncontaminated world soils, which is a point of great concern.

### 4.2. Waters

The surface waters collected from the Regoufe mining area and surroundings are all quite similar in terms of major ions contents (Table 4), being classified as sodium bicarbonated type. Regarding the physicochemical parameters, these waters can be considered circumneutral and evince low EC values in both sampling campaigns (Table 4). The concentrations of the most concerning PTEs are low (only metal(loid)s with high ratios between soil concentrations and GB_local_ are presented; tungsten concentrations were not presented due to its low mobility under local conditions). In fact, only a few samples exceed the Portuguese As and Cd maximum admissible levels for drinking waters, whereas the concentrations of all the analysed PTEs are far below the thresholds for irrigation waters (Table 4), which, in this case, is the only function these waters are destined for. Given these physicochemical and chemical characteristics, all water samples can be considered nearly-neutral and low-metal waters, according to the Ficklin mining-impacted waters classification [51]. These results are quite similar to those obtained by Correia et al. [52] and suggest that Regoufe waters do not represent a case of acid mine drainage, which is a typical phenomenon of mining areas with the occurrence of sulphides [53,54]. However, Favas and Pratas [55] reported cases of acid mine drainage in the Regoufe mine area, with considerably higher metal(loid) concentrations. Nevertheless, the values reported by these authors were still below the national guidelines for irrigation waters. In addition, it was also stated that stream waters under the mine influence have chemical compositions between acid mine drainage and uncontaminated stream waters; thus, the recorded hydrochemical differences likely reflect seasonal fluctuations.

Therefore, surface waters of the Regoufe area do not show cases of severe contamination, despite the high concentrations of several metal(loid)s in soil samples. This can be explained, in part, by the circumneutral pH values, which do not promote the dissolution of PTEs, as well as by the secondary precipitated mineralogical phases and SOM that seem to be effective receivers of the metal(loid)s released from ore minerals. In addition, the attenuation of contamination processes promoted by the long period since the end of mining activities contribute, undoubtably, to a decrease in PTEs transfer to and between environmental compartments at the surface, as it stops the input/remobilisation of metal(loid)-rich materials on the surface.

## 5. Conclusions

Mining and beneficiation processes at the abandoned Regoufe mine have produced arsenopyrite-rich mine wastes that are responsible for the high levels of metal(loid)s found in the area.

The geochemical data allows one to verify that soils from the Regoufe region are enriched in some PTEs in relation to the geochemical background of Portuguese soils and to uncontaminated world typical values. However, and despite this enrichment, soil samples showed unusually high contents of several metal(loid)s, in particular As, Bi, Cd, Mn, Pb, Sb, U, W and Zn, denoting a contribution from ore-processing activities, which resulted in relevant local environmental impact. In fact, about 67% of topsoil samples show a high to ultra-high degree of contamination, as demonstrated by the *m*C*_d_* calculus. Notwithstanding, it is for As, Cd and W that average amounts exceed, respectively, 55, 12 and 15 times the already high local geochemical background values, representing a remarkably high level of contamination, as shown by their respective CF values. Arsenic and Cd are undoubtedly the PTEs of most concern due to their inherent toxicity and high mobility, while W toxicity is not well known and mobility is low in acid environments, which are the prevailing conditions in these soils (pH median value of 4.30).

Spatially, the highly contaminated area is circumscribed along a NW–SE subcircular region around the mine adits, appearing to be strongly influenced by arsenic distribution, since it is the PTE showing higher concentrations in the soils. Although geomorphology greatly limits the main hotspot of contamination, the partially shielded agricultural soils are still characterised by high levels of arsenic, which may represent a risk to human health if this metalloid enters the food chain. Although sequential selective chemical extraction results showed that Cd and As are mainly retained in Fe-oxyhydroxides and organic matter, the amounts (concentration values) of As removed from the F1 (bioavailable fraction) and F2 (acid-soluble forms) steps—available support phases—are considerably high, even with quite low extraction percentages.

After all, the amounts of PTEs in surface waters were low and substantially below the admissible levels for irrigation waters, although As and Cd can punctually surpass the drinking water standards. The circumneutral pH values of these waters and their sequestration in secondary mineralogical phases and in organic matter do not promote metal(loid) dissolution, which may explain their low concentrations in surface waters of this area.

Finally, this work is intended to be a decision support tool, aiming at highlighting the most critical areas that need intervention, so that risks to local populations and ecosystems can be minimised and contaminant migration can be limited.

## Figures and Tables

**Table 1:** Descriptive statistical summary of the analysed physicochemical parameters and chemical concentrations of selected elements from the topsoil samples of the Regoufe area (*n* = 61). Local geochemical background values (GB_local_) are an average between those determined for samples collected on granitic (GB_gr_) and metasedimentary (GB_m_) bedrocks (see text).

Parameter	pH_CaCl2_	EC	SOM	CEC	Fe	Mn	As	Bi	Cd	Cu	Pb	Sb	U	W	Zn
Unit		μS cm^−1^	%	cmol^+^ kg^−1^	%	mg kg^−1^									
Minimum	3.40	7.80	0.14	0.16	0.73	239	10.2	2.50	0.06	1.6	14.1	0.64	3.40	11.1	66.5
Median	4.30	29.2	7.6	16	1.4	755	1113	11.7	1.5	13.0	54.4	2.8	13.8	119	172
Maximum	6.00	158	49	48	7.5	1374	23,876	183	32	134	597	40	89.9	2226	1320
Mean	4.34	43.2	9.5	17	2.1	787	2560	23.3	3.6	25.0	83.9	5.9	16.7	199	238
SD	0.48	35.5	8.7	12	1.6	281	4948	35.9	6.0	26.7	101	7.9	13.5	359	211
GB_local_	-	-	-	-	4.5	387	46.4	4.00	0.30	27.5	36.9	1.1	4.62	13.7	140
GB_gr_	-	-	-	-	0.89	530	50.5	4.00	0.20	4.2	24.7	1.5	5.90	23.1	86.8
GB_m_^1^	-	-	-	-	8.2	244	42.2	N.A.	0.39	50.9	49.2	0.69	3.34	4.2	193
World ^2^	-	-	-	-	3.5	488	6.8	0.42	0.41	38.9	27.0	0.67	3.00	1.7	70.0
Portugal ^3^	-	-	-	-	2.4	481	15.0	0.40	0.30	18.6	19.0	1.6	3.00	1.0	50.6

EC: electrical conductivity; SOM: soil organic matter; CEC: cation exchange capacity; SD: standard deviation; N.A.: not available; ^1^ values from Fonseca [38]; ^2^ world soil averages from Kabata-Pendias [39]; ^3^ continental Portugal soil averages from Ferreira [40].

**Table 2:** Eigenvalues and eigenvectors of the principal components.

Principal Component	PC1	PC2	PC3
Eigenvalue	7.00	5.10	2.12
Proportion in total variance	38.50	25.50	10.61
Cumulative proportion	38.50	63.99	74.60
**Eigenvector (loading)**			
Al	−0.50	0.25	0.41
Ca	0.17	0.12	0.33
K	−0.56	0.72	0.12
Mg	−0.03	−0.83	0.47
Na	−0.27	0.63	−0.19
P	0.37	0.02	0.77
Ti	−0.09	−0.88	0.29
Fe	0.73	−0.57	0.14
Mn	0.37	0.71	0.44
W	0.79	0.25	0.01
Sn	0.01	0.85	0.15
As	0.93	0.05	−0.26
Be	0.23	0.66	−0.24
Bi	0.89	0.06	−0.29
Cd	0.91	0.15	−0.02
Cu	0.85	−0.41	0.09
Pb	0.88	−0.03	−0.27
Sb	0.92	0.17	0.05
U	0.23	0.55	0.56
Zn	0.81	0.26	0.27

**Table 3:** Percentage distribution of soil samples according to the degree of contamination defined by the *m*C*_d_* index.

Samples (%)	*m*C*_d_* Value	Contamination Classes
4.9	*m*C*_d_* < 1.5	None to very low
1.6	1.5 ≤ *m*C*_d_* < 2	Low
26.2	2 ≤ *m*C*_d_* < 4	Moderate
29.5	4 ≤ *m*C*_d_* < 8	High
27.9	8 ≤ *m*C*_d_* < 16	Very high
1.6	16 ≤ *m*C*_d_* < 32	Extremely high
8.2	32 ≥ *m*C*_d_*	Ultra-high

**Table 4:** Descriptive statistical summary of the analysed physicochemical parameters and chemical concentrations of selected ions/elements from surface waters collected in the Regoufe area (*n* = 7 (wet season) and *n* = 3 (dry season)).

	Parameter	pH	EC	Cl	HCO_3_	SO_4_	Ca	K	Mg	Na	As	Cd	Mn	Pb	Sb	U	Zn
	Unit		μS cm^−1^	mg L^−1^							μg L^−1^						
Wet season	Minimum	5.99	19.2	3.7	5.92	1.0	1	0.7	0.2	0.2	0.78	0.04	1.04	0.21	0.01	0.001	19.6
	Median	6.11	24.2	3.9	11.8	2.4	2.4	1	0.2	0.3	36.8	0.76	8.51	0.46	0.08	0.25	71.0
	Maximum	7.02	28.8	4.5	13.8	3.2	3.2	1.6	0.3	0.7	134	2.88	13.6	0.98	0.54	0.57	144
	Mean	6.25	24.4	4.0	10.7	2.2	2.2	1	0.2	0.4	49.9	1.25	8.08	0.51	0.14	0.24	72.6
	SD	0.35	3.48	0.27	2.51	0.78	0.78	0.3	0.05	0.24	58.0	1.13	4.87	0.27	0.19	0.24	47.9
Dry season	Minimum	6.61	34.5	1.2	19.7	0.8	1.3	0.4	0.6	2.8	2.37	0.4	11.1	0.13	0.01	0.01	25.9
	Median	6.66	46.0	4.3	19.7	4	1.9	0.8	0.7	3.9	16.8	0.75	12.5	0.24	0.02	0.01	40.8
	Maximum	6.88	51.5	4.7	19.7	4.6	2.6	0.9	1.2	3.9	206	9.45	12.5	0.51	0.36	0.64	484
	Mean	6.72	44.0	3.4	19.7	3.1	1.9	0.7	0.8	3.6	75.0	3.53	12.0	0.29	0.13	0.22	184
	SD	0.14	8.67	1.92	0	2.04	0.63	0.3	0.28	0.63	114	5.13	0.82	0.19	0.2	0.36	260
Guideline values	Drinking water ^1^	6.5–9.5	2500	250	N.A.	250	100	50	12	200	10	5.0	50	10	5.0	N.A.	3000
	Irrigation water ^2^	4.5–9.0	1000	70	N.A.	575	N.A.	N.A.	N.A.	N.A.	10,000	50	10,000	20,000	N.A.	N.A.	10,000

EC: electrical conductivity; SD: standard deviation; N.A.: not available; ^1^ maximum admissible values for drinking waters according to Portuguese legislation [56,57]; ^2^ maximum admissible values for irrigation waters according to Portuguese legislation [56].

## Data Availability

Data is contained within the article. For detailed information of each individual sample, please contact the corresponding author.
